# What a wish to die can mean: reasons, meanings and functions of wishes to die, reported from 30 qualitative case studies of terminally ill cancer patients in palliative care

**DOI:** 10.1186/1472-684X-13-38

**Published:** 2014-07-31

**Authors:** Kathrin Ohnsorge, Heike Gudat, Christoph Rehmann-Sutter

**Affiliations:** 1 Hospiz im Park, Hospital for Palliative Care, Stollenrain 12, CH-4144 Arlesheim, Switzerland; 2 Institute for the History of Medicine and Science Studies, University of Lübeck, Königstrasse 42, D-23552 Lübeck, Germany

**Keywords:** Wish to die, Wish to hasten death, Desire to die, Meaning, Aetiology, Ethics, Palliative care, Cancer, Oncology

## Abstract

**Background:**

Despite research efforts over recent decades to deepen our understanding of why some terminally ill patients express a wish to die (WTD), there is broad consensus that we need more detailed knowledge about the factors that might influence such a wish. The objective of this study is to explore the different possible motivations and explanations of patients who express or experience a WTD.

**Methods:**

Thirty terminally ill cancer patients, their caregivers and relatives; from a hospice, a palliative care ward in the oncology department of a general hospital, and an ambulatory palliative care service; 116 semi-structured qualitative interviews analysed using a complementary grounded theory and interpretive phenomenological analysis approach.

**Results:**

Three dimensions were found to be crucial for understanding and analysing WTD statements: intentions, motivations and social interactions. This article analyses the *motivations* of WTD statements. Motivations can further be differentiated into (1) *reasons*, (2) *meanings* and (3) *functions. Reasons* are the factors that patients understand as causing them to have or accounting for having a WTD. These reasons can be ordered along the bio-psycho-socio-spiritual model. *Meanings* describe the broader explanatory frameworks, which explain what this wish means to a patient. Meanings are larger narratives that reflect personal values and moral understandings and cannot be reduced to reasons. *Functions* describe the effects of the WTD on patients themselves or on others, conscious or unconscious, that might be part of the motivation for a WTD. Nine typical ‘meanings’ were identified in the study, including “to let death put an end to severe suffering”, “to move on to another reality”, and – more frequently– “to spare others from the burden of oneself”.

**Conclusions:**

The distinction between reasons, meanings and functions allows for a more detailed understanding of the motivation for the WTD statements of cancer patients in palliative care situations. Better understanding is crucial to support patients and their relatives in end-of-life care and decision making. More research is required to investigate the types of motivations for WTD statements, also among non-cancer patients.

## Background

Despite research efforts over recent decades to improve understandings of why terminally ill patients express a wish to die (WTD), there is broad consensus regarding the need for more detailed knowledge about the factors that might influence the occurrence or the intensity of a WTD [1]. Early studies [2-6] of the aetiological factors underlying WTD statements highlight the association between a WTD and depression or hopelessness. These studies were conducted with relatively large patient samples (N = 100-378), but the self-report questionnaires used often did not allow for in-depth insight into the patients’ narratives, attitudes, thoughts or their psychosocial and spiritual backgrounds (but see Chochinov et al. [5]). Over the last decade, a number of prospective qualitative studies [7-15] have provided deeper insight into patients’ experiences, attitudes and moral beliefs. Most studies report a *multifactorial aetiology*, with physical symptoms (if well controlled) considered less influential than psychosocial and existential-spiritual distress, such as depression, demoralisation, hopelessness, spiritual abandonment, fears about the future, and the fear of losing control or one’s sense of self. Recent reviews [1,16-19] confirm that WTD statements have a multifactorial aetiology and suggest that patient decision making is considerably determined by psychosocial, existential and spiritual factors or beliefs, such as the perception of being a burden to others, poor family cohesion or social support, high levels of anxiety or symptoms of depression, existential suffering, feelings of hopelessness, loss and demoralisation, and lower levels of religious belief. Several studies show that the intensity of somatic symptoms such as pain has only a limited impact on the wish to hasten death [9,14,20-23].

Even though there is a certain amount of evidence for various aetiological factors, there is a lack of insight into the significance of a WTD in the individual context, as seen from the perspective of patients themselves. We need a deeper and more nuanced understanding of what patients mean when they express a WTD. For example, patients can mean different things when they express a fear of ‘losing autonomy’: is it a fear of becoming physically dependent on life-supporting technology, of finding themselves in a situation of social dependence, of losing the possibility of meaningful activity, or of being exposed to hospital routines? Or is the patient expressing a desire to preserve self-determination in terms of planning what happens during the last moments of her or his life? Similarly nuanced meanings can be expected for other triggering factors, such as the fear of being a burden to others, a perceived loss of dignity or a loss of meaning in life. This suggests that empirical findings, even if self-reported by patients in survey studies, have only limited explanatory value if they are not complemented by deeper understanding of the individual person’s views on these statements, something that requires qualitative methodologies. It has been acknowledged that among some patients, a WTD persists even with access to the best palliative care [24]. However, talking with patients about their WTD statements is still perceived as challenging and is therefore often avoided by caregivers [25].

We report on a qualitative interview study involving 30 terminally ill cancer patients, their families and healthcare givers in Swiss palliative care settings. In Switzerland, assisted suicide is legally permitted under the premise that assistance, which can be provided by any – also a non-medical – person, is not motivated by selfish reasons. Suicide assistance in Switzerland is usually provided by private, non-profit, right-to-die organizations based on volunteer work [26]. Culturally, the strong liberal tradition in the country shapes public attitudes towards and acceptance of the right to die [27]. However, while assisted suicide is legally permitted, euthanasia is not. This Swiss context provided the legal and cultural framework in which the interviewees for this research talked about the WTD.

The findings show that a WTD is a complex subjective and social phenomenon, a process rather than a mental state [28]. Data analysis revealed similar patterns for all WTD, namely that they are constituted of three elements: intentions, motivations and constitutive social interactions. In the following, we describe our findings on the second element: ‘motivations’. This addresses the question of why – from the perspective of patients – a WTD is present. Since why-questions can bear multiple meanings, the ‘motivations’ category has been subdivided into ‘reasons’, ‘meanings’ and ‘functions’. To make this particular model comprehensible, we first briefly explain what we call the ‘contextual anatomy of a WTD’, which was developed on the basis of the study findings.

## Contextual anatomy of a wish to die

In the descriptions of WTD that we heard in the interviews with patients, relatives and caregivers, and which we systematically analyzed, we saw different layers and dimensions that together compose a wish. In iterative comparison with the empirical data and through a series of discussions among the research team, we developed a clarified theoretical model of WTD, which can account for the findings in the data.

The focus of this paper is on the motivations that patients can experience as underlying their WTD. ‘Motivations’ for a WTD can be distinguished from intentions and social interactions, which are also constitutive elements. Motivations appear to consist of three layers: reasons, meanings and functions.

A wish cannot be modeled like an organism, with the content and workings of its inner parts describable through dissection and functional analysis. It necessarily consists of relations to persons and things other than the wisher. We therefore present here a ‘contextual anatomy’ of a WTD. The model was not pre-conceived as an analytic tool *ex ante* in preparation for the fieldwork, or in order to interpret the data, but is itself the result of intensive exchange when going back and forth between the empirical data, our attempts to make sense of the data and also our theoretical knowledge of the current literature.

The model describes the various dimensions that we all found relevant for the WTD statements in our study. Clarifying these dimensions allows for a better understanding of a patient’s WTD while also supporting more appropriate care.

**Figure 1:**
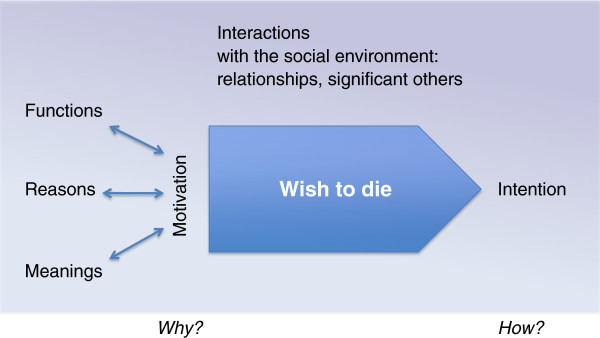
A wish to die: its motivation, intention and the constitutive social interactions.

*Definitions:* We define a *‘wish to die (WTD)’* as the wish for death to come; a *‘wish to live’* as a wish for life to continue; and *‘acceptance of dying’* as holding an accepting attitude towards one’s own dying. Acceptance is not regarded as a wish, since the volitional component is lacking, something that is essential for a wish.Data analysis revealed a pattern whereby WTD were constituted of three dimensions: (1) intentions, (2) motivations and (3) social interactions between the patient and other persons (Figure 1).

### The intention of a WTD

The intention reveals how someone wishes to die: for instance, by wishing to hasten or not to hasten death, by actively contributing to its coming sooner, etc. Intentions can change over time and they can coexist alongside one another (even with wishes to live, as we have pointed out in [28]). In WTD statements, we found nine different types of intention (see below), which can be clustered into three groups: a) wishes to die without the wish to hasten death, b) wishes to die where patients consider hastening death without undertaking actions that lead to it, and c) wishes to die where patients undertake actions to hasten death. A wish to die, therefore, does not necessarily imply that a person intends to hasten death, or that the person will act out her or his wish.

Patients’ statements expressing their wishes about the end of their life can fall into one or more of the following intentions towards dying (first published as a table in [28]):

Wish to live

Acceptance of dying

Wish to die:

Not considering hastening death

1. Looking forward to dying

2. Hoping that dying happens more quickly

3. Desiring to die (but hastening death is not considered)

Considering hastening death

4. Hypothetically considering hastening death (in future, if certain things happen)

5. Actually considering hastening death, but at the moment (for moral or other reasons) it is not an option

6. Actually considering hastening death, hastening death is a (moral) option

Will to die

7. Explicit request

8. Refusing life-sustaining support (such as food or treatments) with the intention of hastening death

9. Acting towards dying (such as suicide or assisted dying)

### Motivations for a WTD

The underlying or overt motivations explain, in the subjective view of the patient, *why* a WTD is present. It is useful to distinguish between three different components of the motivational complex: reasons, meanings and functions. *Reasons* are the factors that patients understand as causing them to have or accounting for them having a WTD. These can be different things, such as particularly burdensome events in their lives, symptoms like pain or fear, social circumstances such as loneliness, financial shortage, lack of a care network, or being in spiritual need. The reasons, as they are possible to include in this model, can be ordered along the bio-psycho-socio-spiritual model, which is widely used in palliative care practice. For most patients in this study, their WTD was insufficiently explained by reasons alone. Patients also saw their WTD to signify something, which explained what this wish meant to them. What patients referred to in this respect we call the *meanings* of a WTD. These are larger narratives that reflect personal values and moral understandings. Some WTD and their statements seem to have a *function* or functional effect, either within the patients’ internal emotional world (for instance, to re-establish a sense of autonomy) or regarding other people and relationships (for instance, to prompt a more serious conversation with caregivers). These effects on the self or on others, conscious or unconscious provoked, were for some patients a further component of the motivational complex of their WTD.

### Social interactions constituting a WTD

WTD statements are situated in the local socio-cultural contexts where patients are socialised. Within these sometimes changing settings, social relationships influence and co-constitute a particular WTD. This can happen either through direct or indirect communication, through the assumed or real reactions of others or through dominant cultural schemes. The act of declaring a WTD to others often changes something in the corresponding relationship, which in turn has a further effect on a patient’s WTD. How others understand the patient’s wishes morally (or what the patient assumes that others believe in moral terms) also contributes to how a wish is interpreted by the patient (more on this in [29]). There may be some overlap between social interactions and functions, since the latter are also socially interactive. The key point in the category ‘functions’ is that the activity starts from the patient’s side and that the WTD is a means to cause an effect on others, or to act as an instrument like an appeal or a trigger to induce friction. The key point in the category ‘constitutive social interactions’ is that the interaction brings the WTD about.

## Methods

We used a qualitative research methodology that combined phenomenological and hermeneutic approaches and was mainly inspired by Interpretive Phenomenological Analysis [30], but also by Grounded Theory [31]. Descriptions of the philosophical concepts underlying this approach have also been given elsewhere [28,32]. This approach is conceived of as being as open as possible to the participants’ subjective views and experiences and refrains from judging a patient’s views. In particular, the interviewers avoided moral judgments about controversial end-of-life practices. Similar methodologies were tested in previous studies [33,34]. Prior to the main study, the interview schedule was tested in a pilot study with 5 patients, their relatives and caregivers.

### Sampling

Patients with advanced cancer disease were asked to narrate their ideas and wishes regarding living and dying. Included in the sample were inpatients in a palliative care hospital specialized in end-of-life care, a palliative care ward within the oncology department of a general hospital, and outpatients of an ambulatory palliative care service, all in the region of Basel, Switzerland (see Table one in [28]). Inclusion criteria for patients were: patients (i) with incurable cancer in (ii) a palliative situation (characterized by limitations of antitumor treatment, predominant use of palliative measures, limited life expectancy), who (iii) had been informed about the incurability of their disease, (iv) were cognitively in a condition to be interviewed, (v) whose primary physician had agreed to their enrolment in the study, and who (vi) consented to participate. Each patient was tested for depression (anamnesis and screening using Robinson’s mini-screen for depression [35], and in cases of assumed depression, additionally using the Beck-Depression-Inventory [36]). Only severe depression was a criterion for exclusion, since the interview might have led to an unmanageable situation.

Patients were selected as far as possible through theoretical sampling [31], though this was sometimes limited by factors of convenience due to the low number of cases that met all inclusion criteria. Some convenience factors are explained by the difficult interview conditions, as we were interviewing persons who were exceptionally close to dying. The median interval between the final interview and a patient’s death was 22.5 days (range 5–237 days, with 2 patients not included since they were not considered to be in a terminal state when they entered the study and were still alive at the end of the study period).

Thirty-two out of 34 identified patients agreed to participate in the study. Two declined because speaking was too burdensome. Two patients were excluded retrospectively because they were not compatible with the inclusion criteria. Thirty case studies of patients, their caregivers and relatives were analysed (total 116 interviews). Eight patients were interviewed more than once. In 18 cases, one of the patients’ close relatives was interviewed, as selected by the patients. Four patients had no relatives to be interviewed, 5 patients refused any interviews with relatives (because of conflicts with relatives or the high burden already on relatives), and in three cases interviews were refused by the relatives (too burdensome). All patients gave informed consent for us to collect and analyse their medical records and to interview their caregivers. In all cases, at least one physician (hospital or family doctor) and/or one nurse were interviewed.

Three out of 30 patients suffered from depression, one with a borderline syndrome. At the time of the interview, all depressive patients were taking antidepressants. In no cases was the patient’s decision making capacity compromised by depression.

### Practical approach to patients and data collection

The study was introduced to the patients by the attending physician. The risk of selection bias was accepted, since protection of particularly vulnerable patients was considered more important. It was explained to participants that the aim of this study was to investigate the experiences of patients with severe illness, what they need and what their ideas are regarding living and dying. Interested patients were then visited by the interview team in order for them to introduce themselves, to clarify questions and to obtain written informed consent. Participants were interviewed face-to-face at the place of their choice, mostly at the place where they received medical care. Patients and relatives were interviewed by two trained interviewers who were not involved in the patients’ care or treatment. The interview team consisted of an interdisciplinary group of interviewers all working in the field of palliative care (one bioethicist, one art therapist, two palliative care nurses, one pastoral care worker and one palliative care physician). Training was obtained in the preceding pilot study.

Interviews lasted between 30 and 90 minutes. Non-participants were not present during the interviews. Interviews were semi-structured, leaving much space for the participants’ own narrations. We started with a schedule but probed important topics as they arose. Central questions were: “In the course of your illness, did you ever have the wish that your disease would proceed more rapidly?”, “Can you imagine situations in which you would prefer not to continue living?” or “Did you ever think of putting an end to your life?” The term “wish to die” was only used when introduced by the patient. The interview schedule contained additional questions, so that only participants who said things that could be related to a WTD were posed questions regarding such wishes. Those who did not express any ideas close to a WTD were not asked further about the issue. Patients with a WTD were asked to describe their perceptions of triggering or hindering factors, relational aspects, the importance of autonomy and spirituality, and also whether the permissive situation of organized assisted suicide in Switzerland influenced their attitude. All interview questions were asked in a way to call forth personal meanings and moral understandings. The interviews, however, carried no special interest in probing assisted suicide per se.

In accordance with our methodology [30,37,38], the interview guide was continuously refined on the basis of the experiences gained from the interviews. All interviews were conducted in Swiss-German idioms, audio-recorded, and subsequently fully transcribed in German using simple verbatim transcription rules. For publication, the selected interview quotes have been translated from German to English.

The interviews with nurses, doctors and relatives took place either on the same day or a few days after the patient interview and were centered on the experiences and ideas of the patient. They lasted between 20 and 60 minutes and were conducted by one interviewer. Adapted schedules were used in these interviews. We wanted to know how these groups saw patients’ wishes regarding death, to what extent relatives and caregivers were informed about these ideas, and which conversations, interactions or reactions were important with regard to a patient’s WTD statement.

### Ethics

We encouraged patients to interrupt or postpone the interviews if they felt emotionally burdened or demonstrated signs of tiredness. Interviewed patients and relatives were personally followed up by the primary physician shortly after the interview and checked for adverse effects (which were reported by only one patient participant who was strongly affected by childhood memories). No content from other interviews was disclosed to the patients, relatives or caregivers, since this would have breached confidentiality and could have influenced the relationship and ongoing discussions about the issue. The study was approved by the ethics committee of Basel (Ethikkommission beider Basel).

### Analysis

Transcripts were continually analyzed during the interview period. The idiographic approach of Interpretative Phenomenological Analysis is dedicated to generating in-depth psychological knowledge of subjective experiences and personal meanings [30]. Grounded Theory enables analysis of the data on a higher sociological level of abstraction [38]. We used both approaches complementarily. After initial independent coding of the transcripts by each of the authors, the coding and interpretation of all interviews belonging to one case unit (interviews of one patient, his/her relative, his/her nurse and physician) were discussed by the three authors for each patient story. In case of disagreement in interpretation, we discussed the issue until we had gained a deeper understanding and mostly also a common interpretation. The discussion of our separate interpretations also led to a list of emerging themes for each case unit (higher order themes with several sub-themes). Apart from the ‘set themes’ (stemming from the initial research questions) in the interview schedule, we searched for and characterized ‘emerging themes’. After 14 interviews, a provisional list of emerging themes was compiled, which was then used together with the list of the ‘set themes’ to support further data analysis and was subsequently finalized. The interpretations of clusters of case units developed by the authors were then critically discussed at group meetings, which also included the interviewers.

Thematic theoretical saturation was achieved with respect to the general findings and the model that we describe above as the ‘contextual anatomy of the WTD’, as well as with respect to the list of intentions for a WTD [28]. Regarding the ‘meanings’, however, which we report on in detail in this paper, we did not identify sufficient recurring patterns that would allow for theoretical saturation. It is very likely that among non-cancer patients or in other cultural settings, additional or different meanings than those we have found here will be relevant for patients.

In order to highlight meanings in WTD statements, this publication necessarily focuses on the 23 of the 30 patients who were experiencing or had recently experienced some kind of WTD. Patients who stated a clear *wish to live* without a wish to die (N = 5) and those who solely experienced feelings of *acceptance* (N = 2) have not been included in this analysis. The relatively high number of 23 out of 30 patients with a WTD is explained partly by the theoretical sampling process (as suggested by our methodology [37,38]). Another reason might be that some study patients made WTD statements during the interviews that had not been previously revealed to their caregivers and relatives.

## Results

In the interviews, we focused on subjective explanations that brought patients to express a WTD [20,39]. These ‘motivations’ explain why a WTD was present. Most but not all patients could indicate ‘reasons’ that they held accountable for causing them to have a WTD. The reasons they gave for their WTD referred often to single phenomena or events (pain, particular fears, social isolation, etc.). However, we observed that most often, patients’ motivations for a WTD were not exhaustively explainable by these singular reasons. For most patients, their WTD also had a broader significance, which they brought up when invited to share what this wish meant to them. Patients then responded by explaining their WTD through larger narratives, which reflected their personal values and moral understandings. We call this broader personal significance for the patient the ‘meaning’ of the WTD. While the reasons give insight into what patients themselves see as causing them to have a WTD, the meanings of a WTD reveal what a WTD means within the patients’ self-understanding and with respect to his or her personal values. In addition, some wishes seem to be expressed, consciously or unconsciously, to achieve a certain effect, either within the patient’s internal emotional world or on other people. This type of motivation, which was more rare, we call the ‘function’ of the WTD. In summary, we differentiated between three different motivational aspects: (1) *reasons* for, (2) *meanings* and (3) *functions* of WTD statements.

### Reasons

The reasons that patients saw as causing them to have a WTD were distributed over the entire range of the ‘bio-psycho-socio-spiritual model’ widely used in palliative care [40]. As known from clinical practice, the burden of disease-related bio-psycho, social problems and spiritual needs identified by patients are not necessarily the same as those acknowledged by caregivers. This was also true for our participants.

*Physical* reasons mentioned in the interviews were the experience of acute or chronic pain, suffocation, chronic nausea, incontinence, smelling wounds, ulcerations, drowsiness, etc.

*Psychological* reasons were anxiety, feelings of sadness, loss of perspective and hope, and the fear of being confused, unable to make decisions, becoming dependent on nursing care or being ‘hooked up to machines’.

*Social* reasons included loneliness, social isolation, loss of social role, financial shortage, lack of an adequate caring network, and the experience of having been abandoned by partners or families due to the disease.

P24 explained her WTD in terms of the sudden loss of her income, her home, and her lack of a social network. A significant number of patients said that their fear of being a burden to others was the main reason for their WTD:

P2: “I would like to go. You see, I want to let people off the hook. I don’t, I don’t like it that they always have to… they all have a life too and I don’t want to, that I… well”.

P4 made several strong requests to hasten death because she felt ashamed about her ulcerating and strong smelling tumor, which she assumed was a nuisance to others. P19 gave descriptions of her sporadic WTD, which included the feeling of despair that she was unable to look after her three-year-old son due to fatigue. P5 said that she sometimes wished to die because her treatment choices were not respected by her physicians and she felt exposed to a medical system whose values she only partly shared. She wanted to leave this world because *“I don’t fit into it”*.

*Existential or spiritual* reasons for many patients included the experience of the loss of dignity or activity, the feeling of being locked into a disabled body. Others emphasized the hopelessness and contingency of their current life situation; an awareness of the incurable and terminal condition of their disease, the uncertainty of the dying process and the experience of a profound lack of sense of life.

Patients weighed up reasons differently. Some reasons were true for the past or present, some hypothetically for the future. Life-affirming reasons could exist alongside reasons that led to a wish to die, with and without feelings of ambivalence (see more detailed in [41]).

### Meanings

The 23 patients in our sample with a WTD reported nine different types of meanings. Some of these nine types of meanings appeared more frequently, while others were identified only once. The meanings of a WTD were shaped by personal experiences, cultural background and relationships. The patients’ narratives, their views on important events, breaks and decisions in life, and their disease trajectory all shed light on the values and moral understandings that make up the particular meaning of a WTD. An in-depth analysis of the meanings in the case stories P2 and P7 can be found in [41].

Strikingly, some patients with a WTD (P12, P29) said that they did not see any particular reason that had brought up this wish, though at the same time they could clearly explain the meanings this WTD had for them. P11 said that she was not suffering, but because she knew that she would die soon, she wanted death to come faster (without actually having the wish to hasten death).

We present the meanings we identified in an open typology, which most likely is not exhaustive. For patients with other illness experiences or other cultural backgrounds, their WTD might still have other meanings.

Meanings of wish-to-die statements (open list):

A wish to die can be a wish

1. To allow a life-ending process to take its course

2. To let death put an end to severe suffering

3. To end a situation that is seen as an unreasonable demand

4. To spare others from the burden of oneself

5. To preserve self-determination in the last moments of life

6. To end a life that is now without value

7. To move on to another reality

8. To be an example to others

9. To not have to wait until death arrives

Patients explained their WTD as a wish…

1. ***To allow a life-ending process to take its course (not impeding)***

This kind of WTD is a wish to not impede the process of dying. Patients referred to spiritual ideas, either naturalistic or religious, about life and dying, such as to *“let nature take its course”* (P31) or that *“in the end it is in the hand of God”* (P21). These patients (P17, P21, P31) saw themselves as part of a wider course of events that they did not feel at liberty to interfere with. This meaning was therefore expressed by patients who had a WTD but did not want to hasten death.

P21: “But now I’m just… Now it’s good, now. He [God] can come and get me.

I: Now You [God] can get me [the patient].

P21: (Lets hand fall) Now He *should* get me!”

2. ***To let death put an end to severe suffering (life as a burden)***

Death can be seen as the ‘lesser evil’ and therefore desirable. Several patients (P1, P21, P24, P29, P32) explained that they wished for death to put an end to their severe burden from symptoms or to their existential suffering. In an unbearable situation with no other way out, death was seen as the lesser of two (or more) evils. These patients did not experience life as a burden as such, but their suffering was unendurable and could not be stopped while they continued to live. P1 spoke of the *“indirect pain”* that was provoked by the hopelessness of his physical condition. P29 undertook the first steps of contacting the right-to-die organization EXIT, which offers assisted suicide. She stated:

P29: “It’s horrible, I can tell you. It’s horrible. […] the whole situation.

I: The situation. Not being able to get out of it.

P29: Not being able to get out of it, and every morning the same thing: waking up, being washed, lying there till the evening, the same pain”.

3. ***To end a situation that is seen as an unreasonable demand (imposition)***

Some patients described their WTD as a wish to avoid a situation that they experienced as an affront, an imposition or undignified. P5, P21, P24 and P29 all wished to die in order to bring an end to a situation that they experienced as an unreasonable demand. These WTD statements were often connected to an intensely experienced symptom load plus specific moments or events in which something happened (e.g. a new diagnosis, the breakdown of a relationship, a conflict with a family member), which brought the patient to his or her limit. This could have a moral undertone of being a reproach towards destiny, or towards God. As a result, death was seen as liberation from a situation that was perceived as an affront. The perception of these situations was often bound to the patient’s definition of his or her own identity or social status. For P21, a religious study teacher, it was always important for her to participate in life intellectually. After a long history of brain metastases, her desire to die became concrete. In the interview, she explained that this was because the organ that she perceived as most valuable to her had been affected:

P 21: “Then all the red lights started flashing for me, because it was in your head, wasn’t it. Then I thought: No! No, just no. Now I’ve simply had enough. […] I’ve tortured myself enough; I don’t want to torture myself anymore”.

4. ***To spare others from the burden of oneself (being a burden)***

Patients frequently felt themselves to be a burden to others. P2, P4, P19 and P22 wanted to die in order to unburden their loved ones or caregivers from themselves:

P4: “I am burdened myself, I am such a burden to others; I want to end this”.

Together with this meaning, patients also expressed feelings of dependence on others, of low self-esteem or of shame. Patients felt they were a burden to their close family, to healthcare providers or (financially) to society. Most patients were seriously concerned about people in their surroundings, even though some (P2, P22) knew that they were accepted and willingly cared for.

5. ***To preserve self-determination in the last moments of life (control)***

Among some patients, the WTD was associated with the desire to retain (or regain) control and self-determination in the last period of their lives (P5, P13, P24, P29). Some stated that they would rather die than be subject to a prolonged situation of dependence, exposed to hospital routines, depersonalized care and decision making by doctors on their behalf. All of these patients were members of a right-to-die organization:

P5: “Just out of fear, if I weren’t treated properly”.

For some, preserving autonomy involved concretely planning their end in order to retain control over what would happen to them during the last moments of life. P13’s WTD was hypothetical, but in the weeks before his death he undertook all the necessary steps to be able to die quickly with the assistance of the organisation EXIT:

P 13: “I immediately turned to the option of Exit [pause], because I said I’d like to have this option whatever happens. If things become unbearable for me for some reason, but I’m still not dying, then I’d like to be able to grant myself my own death. And I saw to everything, so that it’s ready, that I have the prescription, and talked to those people. They’re quasi on call now. […] This is really only about ending a situation that has become unbearable, and not having to rely on either being hit by another stroke or some doctor being understanding after all. I want to be able to keep this in my own hands for when the moment comes. I was a very self-determined person all my life, and that’s very important to me”.

6. ***To end a life that is now without value (worthless life)***

For some patients, the WTD was motivated by the wish that death would put an end to a life that was now without value. These patients (P5, P17, P20) experienced their life in this situation as not worth continuing because of a loss of personal relations, of meaningful activity or of dimensions that they considered essential to their identity.

P20: “And I don’t feel this is a life for me [pause], carrying on living like this. That’s why [pause] um, I am [pause] very – how should I put it, so you understand me – I’m on the road, on the move a lot and [pause] then I thought, if I can’t live like before, life has no value, does it? And [pause] I drove my car a lot, and I can’t do that anymore either. […] Yes, did a lot of travelling. […] I feel my life isn’t worth anything at all any more, if I just lie here and wait”.

7. ***To move on to another reality (afterlife)***

For two patients (P6, P16), their wish to die was motivated by their hope for an afterlife. They imagined death as a passage to another form of existence and they said that they were looking forward to what would come afterwards. They did not so much wish that their lives would end as that they would proceed to *“another level of existence”* (P6).

8. ***To be an example to others (teaching)***

Having a positive attitude towards dying can also be a way of instructing others, in the sense of being a good example. P13 expressed the idea of being an example to his children of how dying can be done well. He undertook everything to prepare his death with a right-to-die organization, yet described his WTD as *“still hypothetical”*. For him, the idea of a good death included the right to self-determination, preserving dignity, maintaining open communication within the family, and giving his children time to integrate the loss:

P13: “And that is perhaps the last debt that someone has to pay their children, isn’t it, to make it all possible; first to do with the separation, and second with regard to their own dying”.

His wife confirmed this intention:

“Yes, I think so. He came to hospital with the aim of showing his children how you can die. I mean, with a kind of dignity, perhaps […] Yes. Yes. He said that explicitly”.

9. ***To not have to wait until death arrives (shortening the dying process)***

Wishing to die can also mean wishing to take a shortcut or to avoid a long dying process. Some patients (P11, P12, P30) who were fully aware that they were dying explained their WTD by saying that they did not see much sense in waiting around until death finally came. P12 actually had the wish to hasten his death, but as his son had committed suicide and the family suffered a lot from this, hastening death was morally not an option for him:

P12: “But I’ve been waiting so long for death now. […].

I: Why do you long for it so much, for it to go more quickly?

P12: So that it’s over.

I: So that there’s an end to suffering?

P12: I’m not suffering. But I still have a loving partner and, um, sometimes you say: ‘Better a horrifying end.

I: …than horror without an end’”.

This well known German proverb “Better a horrifying end, than horror without an end”, which the patient starts and the interviewer completes as suggested, embraces the meaning of this WTD as a wish to shorten the dying process in order not to have to wait for death to arrive.

### Functions

Some patients had a WTD or made WTD statements in order to obtain, consciously or unconsciously, an intended effect on themselves or others. Not all WTD statements had a function, but in nearly all of the cases where a WTD had a function, it had first of all a meaning. Only in one case did the WTD statement have a function but no meaning. This was identified as being due to the fact that the patient did not actually have a WTD, but was simply pretending to have one in front of his wife in order to scare and manipulate her (see below); in front of everyone else, he clearly affirmed his wish to live and undertook all steps to stabilize his health as best as possible.

We identified four different functions:

1. ***Appeal***

For some patient narratives, the WTD statement triggered interpersonal interaction or dialogue, or even functioned as a cry for help. In our data, this was the case for patients who were frightened of dying (P19) or who experienced shame or fear of being a burden to others (P2, P22). P22 was a retired army officer without relatives. He requested assistance to die from his general practitioner before coming to the hospice. Under hospice care, his acute WTD changed into a hypothetical one. However, each morning during the rounds he affirmed his belief that he was nothing more than a burden to society, whereupon he was reassured by his physician that he deserved the care after such a long time serving society. His WTD statements served as prompts in order to obtain moral reassurance, which he desperately needed (see also case description Martha, below).

2. ***Vehicle to speak about dying***

Some patients used WTD statements to communicate to others their experiences as they neared death (P2, P4). P19, who clearly had a wish to live, occasionally expressed WTD statements. She said that she could only talk about her death in the third person, as if it were about someone else. In addition to a cry for help in coping with her unbearable physical pain and fatigue, her WTD statements appeared to be a vehicle to enable her to speak about dying.

P 21: “So I was glad that I could talk to him [husband] about it [WTD]. Actually I was the only one, I was able to communicate that and [pause] just be able to let go of the thought, rather than letting it eat into you. Whether you then do it or not is actually secondary. It’s bad for people if they can’t say to anyone: you know, I have thoughts like this sometimes. So I really am glad that I was able to discuss it with him [husband], it did me good as well”.

3. ***Re-establishing agency***

Some patients explained that their WTD functioned as a means to win back some space for agency at a time when their personal agency seemed to be under threat. This was the case, for example, among patients who experienced a WTD in acute situations of distress; e.g., P24 reported having had spontaneous ideas of suicide when confronted with the diagnosis of recurrent cancer while she was without an apartment and lacked sickness and disability benefits. She affirmed, however, that in each instance when she had such thoughts, she knew that she *“wouldn’t have done it”*. We interpreted her WTD statement as having a catalytic function, a way in which to vent her fear and anger and to regain agency.

In the narratives of other patients, the WTD functioned more as a means of reassurance of personal agency (see case description below). This was the case, for example, among patients who expressed a hypothetical WTD.

P13: “…and then this sleepiness and so on, and then at some point at the back of your mind you say: well, how long am I supposed to put up with this? And then it occurs to you: well, you don’t have to, you can get out of it any time. But it’s more of a reassurance […] It’s a reserve”.

4. ***Manipulation***

In some patient narratives, WTD statements seemed to have a manipulative function. Sometimes, WTD statements seemed to be expressed in order to get additional attention from healthcare providers (P2, P19). Other patients said that they expressed WTD statements in a provocative way just to test the reaction of others. As P11 described:

P11: “I’ve also said these tongue-in-cheek things: so, now I’m starting to collect pills. Yes. And then the people concerned, the ones you say that to, they’re shocked, and yet it was said tongue-in-cheek.

I: To test their reaction.

P: Yes, perhaps sometimes a bit of deliberate provocation”.

One patient (P25) with an explicit wish to live frightened his wife repeatedly with his shocking WTD statements. His wife said that his knowledge of her relief that he was not going to commit suicide provided him with a motivation to continue living.

### Interrelations between reasons, meanings and functions of a WTD

Within their larger narratives on the meanings of the WTD, patients sometimes also incorporated the reasons. Nevertheless, reasons and meanings belong to different categories: while the ‘reasons’ refer to what patients experience as causing them to have a WTD, ‘meanings’ refer to how a WTD makes sense for the patient. Some patients, for example, stated that they felt they were a burden (=meaning) because they had concrete financial problems, which made them dependent (=reason). Others suffered from the loss of activity or having an active role in society (=reason) and felt a moral obligation to die soon, as they believed that they had lost the right to exist (=meaning). In other cases, reasons and meanings for a WTD were unconnected. Indeed, two patients did not perceive any reasons for their WTD, but could nevertheless explain it (P11, P12). This does not exclude the fact that these patients might have had reasons that they did not reveal or that palliative care for these patients could not be improved. Rather, it highlights the importance of investigating not just the reasons as reported by patients or the objective triggering factors as observed by medical science, but also of exploring the larger contextual moral understandings connected with the WTD.

Other studies [11] have shown how WTD statements can be used to enable communication or manipulate another person. In our study, even when WTD statements had a function, they usually *also* had a meaning for the patients. In order to take a respectful approach towards patients, we conclude that even a wish that predominantly consists of a function – for example, a cry for help or dialogue – should still be explored in terms of its meanings and subjective reasons. Adequate understanding of an individual WTD statement therefore requires shared dialogue with the patient and detailed insight into the complexity of personal narratives and self-conceptions.

### Case example

Martha (pseudonym), a woman around 80 years, was hospitalised with advanced rectal adenocarcinoma that required palliative care. She insisted on having the option to hasten death with the help of an organisation that provides assisted suicide (EXIT). At the request of the patient, her physician completed a medical report to be sent to EXIT in case she decided to contact them. However, she said that her spiritual beliefs would hold her back: “Because I feel, after you have died, you could be punished for it”. She felt ambivalent. In the course of one discussion with her physician she confirmed her wish to hasten death but also asked fearfully whether her continuous nausea and inability to eat would make her starve to death. She had a complex, highly emotional family situation: although all her family members were members of EXIT themselves they were strictly opposed to her hastening her own death, and expressed their disapproval to her.

Although when completing the medical report this patient undertook concrete steps to make a hastened death possible (intention type 9: “Acting towards dying”), we interpreted her main intention as belonging to type 4: “Hypothetically considering hastening death (in future if certain things happen),” since during the interview she expressed her wish as follows: “When I feel very, very, very wretched, this thought always returns: If you can’t bear it any more, you can actually cut it short. Right at the last I just could [pause]… if it’s even worse than it isnow…”

As reasons for her WTD she indicated her frequent stomach cramping, nausea, incontinence and feelings of shame, and her poor eyesight, which made it impossible for her to read. We interpreted the intention of her WTD to be based on three wider meanings: to let death put an end to severe suffering: see quote in the text under meaning 2; to end a situation that is seen as an unreasonable demand (meaning 3): “It is hard this fate […] it is cruel, I can tell you”; to preserve self-determination in the last moments of life (meaning 5): “I am glad to be still so clear in my head. I still can make my own decisions myself.” Her WTD had also the function of re-establishing agency (function 3): “But it [WTD] is a ray of hope. You can say, if nothing works anymore and things are only getting worse, then you’d still have some way of shortening it.” We also interpreted the emphasis with which she expressed her WTD in part as an appeal for moral support for her own position vis-à-vis her family (function 1).

## Discussion

The distinction between the three aspects of the motivation for a WTD – reasons, meanings and functions – allows for more detailed understanding of the motivations and related experiences, feelings and thoughts that a patient expresses when stating a WTD. On the basis of our analysis, we consider the meanings of a WTD to be at least as important for understanding the wishes of a patient as the causal factors (identified by research) and the reasons (stated by the patients). Even though there is a growing body of qualitative literature on aetiology, research into the meanings of WTD statements is still scarce. In her groundbreaking study, Nessa Coyle [11] described nine possible meanings of a wish to hasten death. Some of the meanings she found correspond to those of our study: her second meaning, “The dying process itself was so difficult that an early death was preferred”, corresponds to our third meaning, “To end a situation that is seen as an unreasonable demand”; her fourth meaning, “A hastened death was an option to extract oneself from an unendurable situation”, corresponds to our second meaning, “To let death put an end to severe suffering”; her fifth meaning, “A manifestation of the last control the dying person can exert”, corresponds to our fifth meaning, “To preserve self-determination in the last moments of life”; and her seventh meaning, “A gesture of altruism”, corresponds to our fourth meaning, “To spare others from the burden of oneself”. However, some of the other meanings that Coyle found we would interpret as ‘functions’. For example, her sixth meaning, “A way of drawing attention to ‘me as a unique individual’”, we have interpreted more broadly as the function of ‘Appeal’, while her eighth meaning, “An attempt at manipulation of the family to avoid abandonment”, compares partly to the function of ‘Manipulation’ that we describe. We nevertheless want to make clear that due to the complexity of subjective experiences and moral meanings, the types of meanings cannot be exhausted by one study alone. Other moral understandings will most likely bring about more or different types of meanings, so far not captured by our or other studies.

Mak and Elwyn [12] describe 5 meanings for euthanasia among cancer patients along a timeline from previous wellness to approaching death, but these remain quite vague: disease progression (which partly corresponds to what we describe under meaning 6); perception of the suffering of oneself and/or one’s significant others (which includes aspects of our meanings 2 and 4); anticipation of a future worse than death (our meaning 2); and the desire for good quality end-of-life care. It is unclear how Mak and Elwyn’s last category, “holding environment”, should be interpreted as a meaning that motivates the desire to hasten death.

Quantitative research necessarily turns patients’ wishes into observable objects. When asked, however, patients, in their attempts to explain what they experience, present their wishes and ideas in a narrative form [30,42]. Narrative theory assumes that self-understanding happens over time, by linking and summarizing several events into a structure in order to make them intelligible to oneself and others [43-45]. This process is necessarily selective and interpretative. The narrative logic through which this linking happens contains and constitutes meaning [46,47]. The meaning of a specific event can only be determined within its proper context and depends on its relationship to other events or ideas important to this person. Narratives contain and constitute self-conceptions [47]; they also contain reasons for action [46]. Philosophical hermeneutics emphasizes that meaning exists prior to articulation and explanation and can sometimes remain inexplicable [48]; on the other hand, meanings are not fixed and abstract but emerge out of our social existence and are partly constructed, defined and revised in dialogue with others [49-51].

WTD statements therefore *necessarily* and not only accidentally take on a narrative form: they summarize and express patients’ thoughts and evaluations of different events (the experience of sickness, physical burden and deterioration, reactions of family members, emotions, spiritual convictions, existential ideas, etc.). For this reason, it seems more appropriate not to reduce WTD statements to symptoms, but to approach them as communicative acts with a history that captures multiple intentions, reasons, meanings and possible functions. To understand a WTD statement requires becoming acquainted with its narrative logic. Short expressions of WTD statements must be linked back to the fuller narrative context, to values and moral understandings, in order to be understandable. An understanding of a WTD has to be verified through interpersonal dialogue. This understanding of the content of a WTD is not possible from an outside observer position but only through mutual dialogue, in which one takes on a normative commitment or obligation by interpreting the patient’s assertions together with her or him.

Progressive incurable disease confronts patients and their families with the vulnerability, fragility and finitude of life. It might be part of a normal coping process that patients balance their motivations for or against life and death, and develop and express a wish to die. This might also hold for patients in good palliative care settings. Although the number of patients in the study does not allow for statistical generalization, we were astonished at the diversity and complexity of the patients’ reasons and meanings behind their WTD.

In clinical practice, it would be important to differentiate between *reasons* and *meanings*, as defined in our model. *Reasons* might be experienced physically as pain and anxiety, and/or as loneliness, deficits in care, having insufficient financial means or as an existential burden. Physicians often limit themselves to searching for *reasons* that can then be *treated*. And indeed, changing certain conditions is of the utmost importance, as it may often lead to a modification of wishes regarding life and death. But a WTD cannot be properly addressed if the *meanings* of the wish remain unexplored. Meanings are loaded with moral beliefs that need to be *understood* and *respected* in communication, disease management and care of patients and their families.

As described in other studies [16,17], the patients in our sample gave predominantly social and spiritual reasons for their WTD. Access to specialized palliative care in our research setting and the (hopefully) good symptom management may be a reason for this. However, it also underlines the fact that even the best palliative care is not able to eliminate ideas or wishes to hasten death. The prevalence of spiritual and social concerns is striking and should be taken into account. Generally, though, we believe that this observation, which has also been made in other studies [1], should not diminish the importance given to physical symptoms.

## Conclusions

Without detailed understanding of the specific intention of a WTD, and without insight into its specific meanings, reasons and functions, it will be difficult to understand *what* a patient actually wants and *why* wishing it is important to her or him. Patients need confidence and trust in order to state what is personally important to them. Clinic professionals should therefore have the appropriate hermeneutic and communicative skills in order to investigate such personal narratives together with the patient. Becoming familiar with the intentions, reasons, meanings and functions of a WTD requires knowledge of its ‘contextual anatomy’. This needs more than just attention and time. Some patients are more articulate if carefully assisted in the formulation of their ideas and their connection to personal values and perspectives. As the content of a WTD can be influenced by conversations about WTD, caregivers have a triple responsibility: first, to cultivate the skill of active listening; second, to reflect on their own ideas and fears; and, third, to facilitate both the patient’s inner dialogue and discussion of his or her wishes about life and dying.

More research is required in order to fully grasp the possible contextual understandings and meanings that patients attribute to their WTD and how they can be influenced by communication with caregivers and relatives. Research is also needed among other (non-cancer) patient groups who face significantly different disease trajectories.

## Competing interests

Heike Gudat is head physician at the Hospiz im Park, Arlesheim. She was interviewed as the attending physician of some of the patients in the sample. In these cases, she did not take part in the data interpretation.

## Authors’ contributions

KO carried out the interviews and drafted the article. HG and CRS conceived the study design, participated in its refinement and coordination, and substantially contributed to the manuscript. All three authors took part in the data analysis. All authors approved the final manuscript.

## Authors’ information

KO is a philosopher and researcher in bioethics (during this project at the University of Basel, now at the Hospiz im Park, Arlesheim, Switzerland). The paper also forms part of her PhD thesis on hermeneutic bioethics, at the University of Amsterdam. Aside from her research focus on ethics in end-of-life care and hermeneutics, her other research interests include clinical ethics, the ethics of long-term care, and feminist and narrative ethics.

HG is a physician for internal medicine with a specialization in palliative care. She is medical head of the Hospiz in Arlesheim. Her special interest lies in psychosocial aspects during end-of-life care. She is actively involved in the *National Strategy Palliative Care* of Switzerland and has a teaching assignment in palliative care at the University of Basel.

CRS is a molecular biologist and philosopher, Professor of Theory and Ethics in the Biosciences at the University of Lübeck, Germany. His research combines qualitative social research and normative reflection in bioethics in end-of-life topics, genomics and transplantation medicine. While directing the Unit of Ethics in Biosciences at the University of Basel, he chaired the Swiss National Advisory Commission on Biomedical Ethics.

## Pre-publication history

The pre-publication history for this paper can be accessed here:

http://www.biomedcentral.com/1472-684X/13/38/prepub
